# Rapid molecular diagnostics of tuberculosis resistance by targeted stool sequencing

**DOI:** 10.1186/s13073-022-01054-6

**Published:** 2022-05-19

**Authors:** Doctor B. Sibandze, Alexander Kay, Viola Dreyer, Welile Sikhondze, Qiniso Dlamini, Andrew DiNardo, Godwin Mtetwa, Bhekumusa Lukhele, Debrah Vambe, Christoph Lange, Muyalo Glenn Dlamini, Tara Ness, Rojelio Mejia, Barbara Kalsdorf, Jan Heyckendorf, Martin Kuhns, Florian P. Maurer, Sindisiwe Dlamini, Gugu Maphalala, Stefan Niemann, Anna Mandalakas

**Affiliations:** 1 National Tuberculosis Reference Laboratory, Eswatini National Health Services Laboratory, Ministry of Health, Mbabane, Eswatini; 2 Baylor College of Medicine Children’s Foundation-Eswatini, Mbabane, Eswatini; 3 Global Tuberculosis Program, Baylor College of Medicine, Houston, TX USA; 4 Department of Global Health, Amsterdam UMC, University of Amsterdam, Amsterdam, The Netherlands; 5 Molecular and Experimental Mycobacteriology, Research Center Borstel, Borstel, Germany; 6 Eswatini National Tuberculosis Program, Ministry of Health, Mbabane, Eswatini; 7 German Center for Infection Research (DZIF), Partner Site Hamburg-Lübeck-Borstel-Riems, Borstel, Germany; 8 Clinical Infectious Diseases, Research Center Borstel, Borstel, Germany; 9 Respiratory Medicine & International Health, University of Lübeck, Lübeck, Germany; 10 The National School of Tropical Medicine, Baylor College of Medicine, Houston, TX USA; 11 Cluster Precision Medicine in Inflammation, University of Kiel, Kiel, Germany; 12 Department of Medicine I, University Hospital Schleswig-Holstein, Kiel, Germany; 13 National and WHO Supranational Reference Center for Mycobacteria, Research Center Borstel, Borstel, Germany; 14 Institute of Medical Microbiology, Virology and Hygiene, University Medical Center Hamburg-Eppendorf, Hamburg, Germany

**Keywords:** Tuberculosis, Drug resistance, Feces

## Abstract

**Background:**

Stool is an important diagnostic specimen for tuberculosis in populations who struggle to provide sputum, such as children or people living with HIV. However, the culture of *Mycobacterium tuberculosis* (*M. tuberculosis*) complex strains from stool perform poorly. This limits the opportunity for phenotypic drug resistance testing with this specimen. Therefore, reliable molecular methods are urgently needed for comprehensive drug resistance testing on stool specimens.

**Methods:**

We evaluated the performance of targeted next-generation sequencing (tNGS, Deeplex® Myc-TB) for the detection of mutations associated with *M. tuberculosis* complex drug resistance on DNA isolated from stool specimens provided by participants from a prospective cohort of patients treated for tuberculosis in Eswatini (*n* = 66; 56 with and 10 participants without *M. tuberculosis* complex DNA detected in stool by real-time quantitative PCR), and an independent German validation cohort of participants with culture-confirmed tuberculosis (*n* = 21**).**

**Results:**

The tNGS assay detected *M. tuberculosis* complex DNA in 38 of 56 (68%) samples; for 28 of 38 (74%) samples, a full *M. tuberculosis* complex drug resistance prediction report was obtained. There was a high degree of concordance with sputum phenotypic drug susceptibility results (κ = 0.82). The ability to predict resistance was concentration-dependent and successful in 7/10 (70%), 18/25 (72%), and 3/21 (14%) of samples with stool PCR concentration thresholds of > 100 femtogram per microliter (fg/μl), 1 to 100 fg/μl, and < 1 fg/μl, respectively (*p* = 0.0004). The German cohort confirmed these results and demonstrated a similarly high concordance between stool tNGS and sputum phenotypic drug susceptibility results (*κ* = 0.84).

**Conclusions:**

tNGS can identify drug resistance from stool provided by tuberculosis patients. This affords the opportunity to obtain critical diagnostic information for tuberculosis patients who struggle to provide respiratory specimens.

**Supplementary Information:**

The online version contains supplementary material available at 10.1186/s13073-022-01054-6.

## Background

Only 7.1 million (71%) of the estimated 10 million individuals with tuberculosis (TB) accessed care in 2019 [1]. A large case detection gap exists for people living with HIV (PLHIV), children, and patients with drug-resistant tuberculosis [1]. Multi-drug resistant (MDR) tuberculosis (resistance to at least isoniazid and rifampicin) now represents 3.3% of new tuberculosis cases and 18% of previously treated cases globally [1] and continues to rise as a proportion of detected tuberculosis cases [2–4]. There is an urgent need for rapid, comprehensive detection of drug resistance of *Mycobacterium tuberculosis* (*M. tuberculosis)* complex strains to guide appropriate treatment regimens [5]. Early identification of patients with multidrug-resistant tuberculosis, rapid molecular drug resistance testing (mDST), and linkage to care is paramount to decreasing transmission of MDR *M. tuberculosis* complex strains.

The etiology of the case detection gap in low and middle-income countries is multifactorial, but in part is due to challenges with sputum collection in children and PLHIV [6, 7]. Young children and PLHIV are often unable to physically provide sputum samples; thus, procedures such as sputum induction or gastric aspiration are required to collect diagnostic specimens for pulmonary tuberculosis [8]. A growing body of evidence demonstrates that *M. tuberculosis* can be found in the stool of patients with tuberculosis. Identification of *M. tuberculosis* complex in stool specimens by polymerase chain reaction (PCR), typically with the GeneXpert® MTB/RIF (Xpert), has demonstrated sensitivity between 60 and 70% against culture on respiratory specimens in children and adults [9, 10]. Hence, stool is now accepted as a diagnostic specimen to detect *M. tuberculosis* complex in children and PLHIV who have difficulty producing sputum [11, 12]. Sensitivity may be improved through specialized DNA extraction protocols [13]. In contrast, stool culture of *M. tuberculosis* complex strains has a sensitivity of under 30% against respiratory culture, limiting the utility of phenotypic drug susceptibility testing (pDST) from stool specimens [14]. Therefore, reliable methods for resistance prediction based on stool specimens are urgently needed.

Herein, we share the results of an investigation to assess the feasibility and accuracy of targeted amplicon-based next-generation sequencing (tNGS) with the Deeplex® Myc-TB assay (Genoscreen, Lille, France) on DNA obtained by a specialized stool DNA extraction method, using an adjusted version of the MP Fast DNA kit for soil (MP Biochemicals, Solon, OH) [13]. We evaluated the performance of tNGS with DNA isolated from stool specimens provided by participants from a prospective cohort of patients treated for TB in Eswatini (*n* = 66; 56 with and 10 participants without *M. tuberculosis* complex DNA detected in stool by real-time quantitative PCR), and an independent German validation cohort of participants with culture-confirmed TB (*n* = 21). We present the first evidence that tNGS not only detects *M. tuberculosis* complex DNA from stool samples in relation to the amount of DNA present, but also provides full mDST predictions for at least 13 anti-tuberculosis drugs.

## Methods

### Study population and setting

For the Eswatini cohort (cohort one), samples were obtained from a prospective study cohort including child and adult tuberculosis patients, at or within two weeks of treatment initiation, and their asymptomatic household contacts. Between 2014 and 2019, outpatients were recruited from tuberculosis clinics at the Mbabane Government Hospital, Baylor Children’s Foundation Clinic in Mbabane, and the Raleigh-Fitkin Memorial Hospital in Manzini. Study data was captured by trained research assistants using uniform case report forms. Respiratory specimens were provided by expectorated or induced sputum in adults and by induced sputum or gastric aspiration in children unable to expectorate. Participants were considered to have confirmed tuberculosis if a respiratory specimen was positive by Xpert or liquid culture with Mycobacteria Growth Indicator Tubes (MGIT, Becton Dickinson, Franklin Lakes, NJ, USA) and probable tuberculosis if radiographs, clinical symptoms and response to therapy were compatible with tuberculosis. For the German cohort (cohort two), stool and sputum samples from adult patients with culture-confirmed pulmonary tuberculosis were prospectively collected, following informed consent for participation in a prospective cohort, at the Medical Clinic of the Research Center Borstel, Germany, between 2018 and 2019. Patients at the Medical Clinic in Borstel, Germany are commonly referred for initiation of TB treatment or diagnostics when the diagnosis is considered at other facilities.

The objective of this study was to assess the feasibility and accuracy of tNGS on *M. tuberculosis* complex DNA isolated from stool in patients diagnosed with tuberculosis. A cross-sectional, convenience sample of specimens with a range of concentrations of *M. tuberculosis* DNA detected by qPCR or negative was selected to evaluate tNGS performance. Furthermore, samples from both cohorts were analyzed for concordance between mDST by stool tNGS and pDST from sputum.

### Laboratory methods

Cohort One: Consistent with Eswatini national guidelines [15], each participant provided two sputum specimens. One was tested by Xpert MTB/RIF (2014–2019) or Xpert Ultra (2019) in accordance with manufacturer instructions [16]. The second specimen was used for culture at the National Tuberculosis Research Laboratory (NTRL) in Mbabane, Eswatini. Sputum cultures were performed with the Mycobacterium Growth Indicator Tubes (MGIT) 960 system, liquid media for the cultivation of mycobacteria, according to manufacturer instructions [17]. Phenotypic DST was performed with the MGIT system for isoniazid, rifampicin, pyrazinamide, streptomycin, ethambutol, and, when indicated, fluoroquinolones, amikacin and capreomycin. Phenotypic DST on solid culture (Löwenstein-Jensen) and for second-line drugs such as moxifloxacin or bedaquiline was not possible to perform due to limitations in laboratory capacity in Eswatini.

Cohort Two: Each participant provided two sputum samples and a stool sample, on the same day as admission for tuberculosis care. Specimens were stained for acid-fast bacilli and analyzed by microscopy and the presence of *M. tuberculosis complex* DNA by Xpert Ultra, if not already performed at the referring hospital. Solid culture (Löwenstein-Jensen) and liquid culture (MGIT) were performed in addition to phenotypic DST for isoniazid, rifampicin, ethambutol, and pyrazinamide. In the case of drug resistance against isoniazid and rifampicin, comprehensive second-line pDST was performed for levofloxacin and moxifloxacin, bedaquiline, linezolid, clofazimine, cycloserine/terizidone, delamanid, amikacin, kanamycin, capreomycin, PAS, and prothionamide (representative for thiamids). Second-line DST was performed in MGIT and interpreted based on World Health Organization (WHO) critical concentrations [18]. For cycloserine, pDST was performed on a solid medium using a critical concentration of 30 mg/L.

For both cohorts, stool was frozen within 12 h of collection at -80 °C. In Eswatini, stool was frozen without preservatives in 2-g aliquots prior to DNA isolation. In Germany, stool was aliquoted with 500 mg stool in one ml 20% Glycerol/PBS. Stool was thawed in batches and DNA was isolated as previously described [13, 19]. In brief, 500 mg of stool was processed using the MP Fast DNA kit for soil (MP Biochemicals, Solon, OH) with a six-minute homogenization via bead-beating disruption on the SI-D238 Disruptor Genie (Scientific Industries, Inc., Bohemia, NY). The isolated DNA was tested with a previously described qPCR [13] or with the Diarella MTB/NTM/MAC kit (Gerbion, Kornwestheim, Germany) following the manufacturer instructions [20] and quantified using H37Rv standard curves.

Isolated DNA was sent to the Molecular and Experimental Mycobacteriology, Research Center Borstel, Borstel, Germany for tNGS analysis. The Deeplex® Myc-TB assay targets full sequences (i.e. coding sequence plus part of promoter region) or the most relevant regions of 18 drug resistance-associated genes (*rpoB*,* ahpC*,* fabG1*,* katG*,* inhA*,* pncA*,* embB*,* gyrA*,* gyrB*,* rrs*,* eis*,* tlyA*,* gidB rpsL*,* ethA*,* rv0678*,* rrl*,* rplC*), combined with genomic targets for mycobacterial species identification (*hsp65*) and *M. tuberculosis* complex strain genotyping (CRISPR locus)^21^. After Deeplex® Myc-TB amplification as instructed by the manufacturer (24-plexed PCR using a single Master Mix), amplicon libraries were prepared using the Nextera XT kit and sequenced with 150 bp paired-end reads using a NextSeq 500 instrument (Illumina, San Diego, California, USA). Analyses were performed using the integrated bioinformatics pipeline v1.3 implemented in the Deeplex® Myc-TB web application [22]. In short, NGS reads were automatically mapped on *M. tuberculosis* H37Rv reference sequences using Bowtie 2 [23], and variants were called with a limit of 3% read proportion depending on coverage depth. Samples were then classified in accordance with breadth of target coverage and categorized by quality as ND, − , + , +  + , or +  +  + . Detected variants were automatically associated with drug resistance or susceptibility, or phylogenetic lineage by comparison with integrated reference variant using the curated ReSeqTB database [21]. When variants were not included in the database, mutations were defined as uncharacterized. Furthermore, a 401-bp segment of the *hsp65* gene is used as a primary reference for mycobacterial species identification [21], the direct repeat region for spoligotype identification of MTBC strains [24], and an internal control sequence to control PCR inhibition. The identification can also be used as control for mixed infections, as not only the best match is reported by the software.

Mixed infection is also signaled by a phylogenetic variant detected at less than 95%, indicating the simultaneous presence of one strain harboring this variant present at this percentage and another strain sharing the same sequence as the reference at this position, present at approx. 100% minus this percentage.

The association between the qPCR cycle threshold category and a successful Deeplex® Myc-TB result was evaluated using a Cochran-Armitage test for trend. Cohen’s kappa statistic was used to compare Deeplex® Myc-TB results on stool to sputum susceptibility results.

The sequencing data has been deposited in European Nucleotide Archive (ENA) database (Accession number: PRJEB47403, https://www.ebi.ac.uk/ena/browser/view/PRJEB47403?show=reads [25].

## Results

### Study cohorts

Cohort one included 66 patients diagnosed with tuberculosis (Table 1); 56 participants with and 10 participants without *M. tuberculosis* complex DNA detected in stool by qPCR. The cohort was predominantly female (59%) with a median age of 31 (Interquartile (IQR 22 to 36) years (10/66 were aged less than 19 years) and 67% were PLHIV with a median CD4 + T cell count of 248 cells/ml (IQR 121–346). The majority had confirmed tuberculosis (96%) with 83% confirmed by Xpert, 73% by MGIT culture on respiratory specimens, and 85% by stool qPCR. The results of respiratory diagnostic testing compared with the stool qPCR are described in Table 2. Among participants positive by stool qPCR, 10/56 (18%) had a concentration of > 100 femtogram per microliter (fg/μl) (approximately 2316 CFU of *M. tuberculosis*), 25/56 (45%) were between 1 and 100 fg/μl, and 21/56 (37%) had < 1 fg/μl of *M. tuberculosis* DNA (approximately 63 CFU of *M. tuberculosis*)^13^.

**Table 1:** Cohort characteristics of participants undergoing stool Deeplex® Myc-TB testing

**Cohort characteristics**		**Eswatini cohort *N* (%)**	**German cohort *N* (%)**
Total number		66	21
Sex	Female	39 (59)	2 (10)
	Male	27 (41)	19 (90)
Age (years)	Median (IQR)	31 (22–36)	30 (22–39)
Age	0 to 18	10 (15)	0
	19 and above	56 (85)	21 (100)
Tuberculosis disease classification	Confirmed	63 (96)	21 (100)
	Probable	3 (4)	0
HIV status	Positive	44 (67)	0
	Negative	22 (33)	21 (100)
CD4(cells/ml)	Median cells/ml (IQR)	248 (121–346)^a^	NA
BMI	Median (IQR)	20 (18–23)^b^	21 (19–24)
Sputum Xpert MTB/RIF Ultra	*M. tuberculosis* complex: Detected	55 (83)	16 (76)
	*M. tuberculosis* complex: Trace	0	2 (10)
	*M. tuberculosis* complex: Not detected	6 (9)	1 (5)
	NA	5 (8)	2 (10)
Xpert rifampicin resistance	Detected	3 (6)	8 (50)
	Not detected	47 (86)	8 (50)
	NA	5 (8)	NA
Sputum culture result	Positive	48 (73)	21 (100)
	Negative	5 (8)	0
	NA	13 (19)	0
Stool PCR qualitative result	*M. tuberculosis* complex	56 (85)	16 (76)
	Detected		
	*M. tuberculosis* complex	10 (15)	5 (24)
	Not Detected		
Stool PCR fg/μl *M. tuberculosis* complex DNA	> 100	10 (18)	2 (13)
	1 to 100	25 (45)	7 (44)
	< 1	21 (37)	7 (44)

*IQR* interquartile range, *NA* not available, *PCR* polymerase chain reaction, *ml* milliliter, *M. tuberculosis complex* Mycobacterium Tuberculosis Complex

^a^*N* = 36, ^b^*N* = 62

**Table 2:** Cross-tabulation of stool qPCR results with respiratory diagnostics (MTB culture and Xpert Ultra) in cohort 1 (Eswatini) and cohort 2 (Germany)

	**Stool qPCR**	
**Sputum Xpert Ultra results by stool qPCR result**		
**Cohort 1**	Positive	Negative
MTB detected	49	6
MTB NOT detected	3	3
Not available	4	1
**Cohort 2**	Positive	Negative
MTB detected	13	5
MTB NOT detected	1	0
Not available	2	0
**Sputum MTB Culture results by stool qPCR result**		
**Cohort 1**	Positive	Negative
Positive	42	6
Negative	3	2
Not available	11	2
**Cohort 2**	Positive	Negative
Positive	15	6
Negative	0	0

**Fig. 1:**
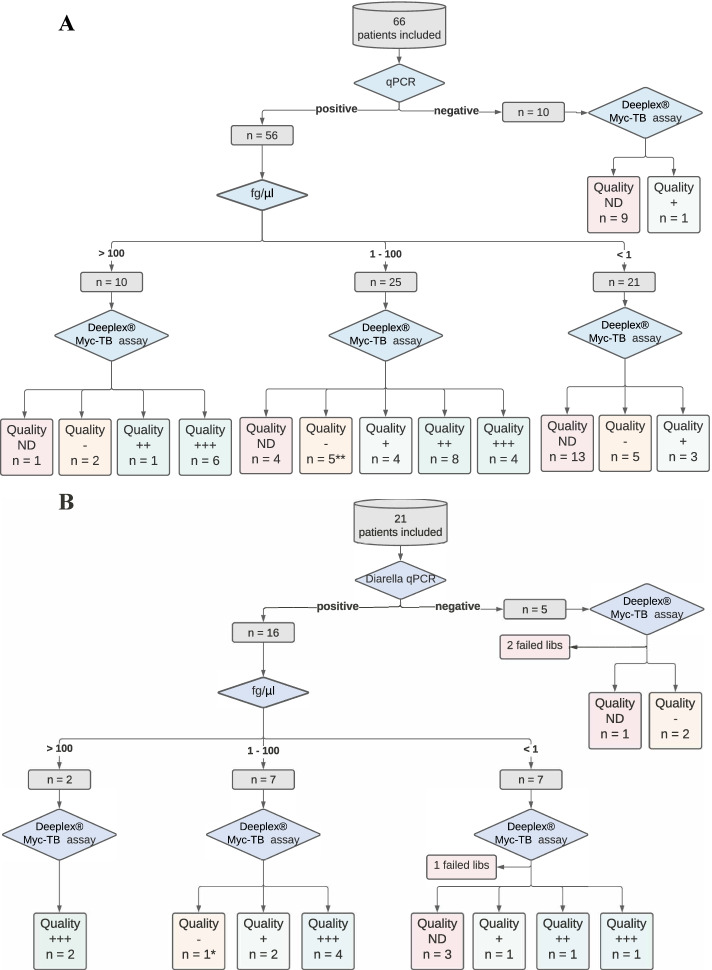
Study flow chart demonstrating results classified in accordance with real-time polymerase chain reaction (qPCR) and concentrations in fg/μl (1 and 100 fg/uL of DNA equates to 62.65 CFU and 2316.13 CFU of H37Rv Mtb/50 mg of stool). Targeted sequencing quality was graded as quality (ND) not detected (red), quality – indicative of partial reads (yellow), and quality + , +  + , and +  +  + indicating complete reads with adequate depth of coverage (green). **A** Cohort 1 and (**B**) Cohort 2. *partial resistance report possible for one sample. Abbreviations: ND, not detected

All 21 participants of cohort two (Table 1) were HIV-negative adults with culture-confirmed tuberculosis, predominantly male (90%) with a median age of 30 years (IQR 22–39). Xpert Ultra detected *M. tuberculosis complex* DNA in sputum of 86% of the patients (16 positive and 2 trace) (Table 2). Of the 16 German participants positive by stool qPCR, 2 (13%) had concentrations > 100 fg/μl, 7 (44%) were between 1 and 100 fg/μl, and 7 (44%) had < 1 fg/μl of *M. tuberculosis* DNA (Fig. 1B).

### Performance of tNGS

Each DNA specimen isolated from stool was evaluated by tNGS. In the Eswatini cohort (Fig. 1A), of ten specimens that were negative by stool quantitative qPCR, tNGS results were negative in nine and positive in one. Overall, tNGS detected *M. tuberculosis* complex DNA in 38/56 (68%) of samples that were positive by *M. tuberculosis* complex qPCR. Of the 38 samples with tNGS results, 28 (74%) had sufficient reads for the prediction of drug resistance in up to 13 anti-tuberculosis drugs.

There was a concentration-dependent relationship for tNGS drug resistance prediction; it was possible for 7/10 (70%), 18/25 (72%) and 3/21 (14%) of samples with stool qPCR concentrations of > 100 fg/μl, 1 to 100 fg/μl and < 1 fg/μl to produce a resistance report, respectively (*p* = 0.0004). This was confirmed by a logistic regression model, which demonstrated a strong association between increasing *M. tuberculosis* DNA concentrations and successful tNGS drug resistance prediction (Additional file 1: Table S1). There was no association between the timing of stool collection within the study enrollment window and successful tNGS (Additional file 2: Table S2). The quality of tNGS results increased with *M. tuberculosis* DNA concentrations, with a median average coverage depth of 2866.1 in samples with qPCR concentrations of > 100 fg/μl, of 1298.4 in samples with qPCR concentrations of 1–100 fg/μl, and 51.1 in samples with a qPCR concentration of < 1 fg/μl.

In cohort two (Fig. 1B), tNGS detected *M. tuberculosis* complex DNA and produced a resistance report in 12/16 (75%) of samples that were positive by *M. tuberculosis* complex qPCR; 2/2 (100%), 7/7 (100%) and 3/7 (20%) of samples with stool qPCR *M. tuberculosis* DNA concentrations of > 100 fg/μl, 1–100 fg/μl and < 1 fg/μl, respectively (*p* = 0.02).

### Detailed resistance analysis

#### Cohort one

**Fig. 2:**
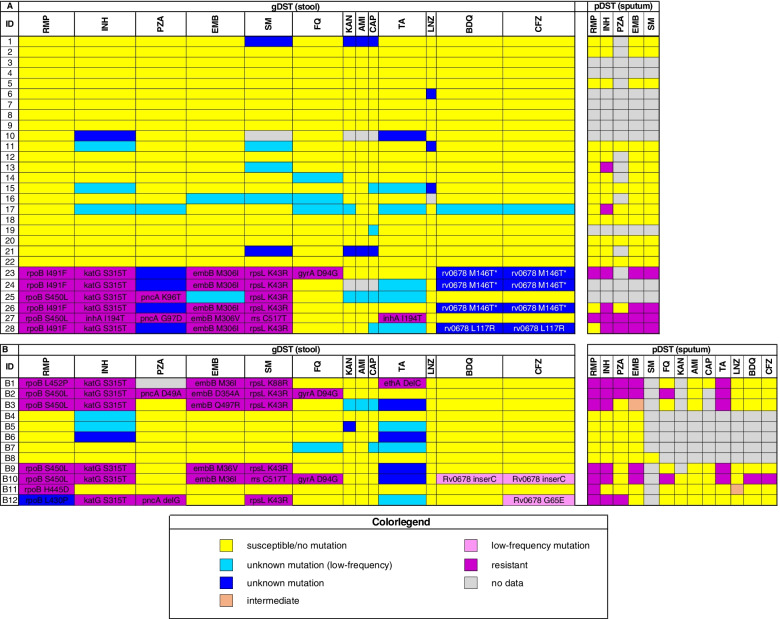
Deeplex® Myc-TB resistotype and pDST resistance detection. Protionamide was tested as a class representative for the thioamides. Mutations associated with resistance are specified in the cells when present. **A** Cohort 1 and (**B**) Cohort 2. Abbreviations: pDST, phenotypic drug susceptibility; RMP, rifampicin; INH, isoniazid; PZA, pyrazinamide; EMB, ethambutol; SM, streptomycin; FQ, fluoroquinolones; KAN, kanamycin; AMI, amikacin; CAP, capreomycin; TA, thionamide; LNZ, linezolid; BDQ, bedaquiline; CFZ, clofazamine

Among cohort one participants with paired tNGS mDST results in stool and pDST results from sputum, there was a high degree of concordance (*k* = 0.82) between the two assays (Fig. 2A and Additional file 1: Table S3). In 18 specimens with paired mDST and pDST results for isoniazid and ethambutol, concordance was substantial to almost perfect (*k* = 0.73 and *k* = 1, respectively). The second-line pDST results for fluoroquinolones, amikacin and capreomycin in participants with first-line drug resistance detected by Xpert or pDST were available in three participants and were concordant with mDST results from stool.

Out of the 28 samples from cohort one (Fig. 2A) with sufficient sequencing quality, six (21%) were classified as multidrug-resistant, four of which harbored the *rpoB* I491F mutation. One sample was classified as extensively drug-resistant based on the identification of a fluoroquinolone resistance mutation and a mutation in *rv0678* [26]; the latter mutation has been defined as a marker for bedaquiline and clofazimine resistance [27]. The pDST and stool mDST on this patient also demonstrated fluoroquinolone resistance. In addition, three other samples with the *rpoB* I491F mutation also had the mutation in *rv0678* and are therefore likely to be bedaquiline and clofazimine resistant. In Eswatini, pDST testing for these medications was not available.

Among the 18 specimens with paired molecular and phenotypic results for rifampicin resistance, two were resistant by both methods, including one specimen identified with the *rpoB* I491F mutation. However, two additional specimens identified with the *rpoB* I491F mutation via mDST were tested susceptible by pDST. The remaining 14 specimens were susceptible by both methods. Overall concordance between mDST and pDST for rifampicin resistance was substantial (*k* = 0.61). Notably, all of the specimens with an *rpoB* I491F mutation also had a *rv0678* M146T mutation that confers resistance to bedaquiline and clofazimine [27].

#### Cohort Two

The German validation set (cohort two) included 21 participants with culture-confirmed tuberculosis. Stool-based mDST results were completely interpretable for drug resistance in 11 of 21 (52%) stool specimens and partially interpretable in one additional sample (B1) (Fig. 2B). Concordance between stool-based mDST with sputum pDST was high (*k* = 0.84) including the drugs used in multidrug-resistant tuberculosis treatment regimens, for which few pDST data were available for the Eswatini samples (Fig. 2B and Additional file 1: Table S3). Six out of these 12 (50%) were classified by mDST in stool as rifampicin-resistant and one sample had an unknown mutation in *rpoB* L430P, which showed resistance by pDST.

Two out of the six samples with rifampicin resistance showed additional resistances: one was classified as pre-XDR-TB based on the identification of a fluoroquinolone resistance mutation and another one defined as XDR-TB due to a combination of fluoroquinolone resistance-mediating mutation gyrA D94G and bedaquiline resistance-mediating mutation in *rv0678*. The pDST on these participants confirmed the genotypically predicted resistances. For clofazimine, mDST in stool identified a *rv0678* G65E mutation, but the corresponding sputum specimen was determined to be susceptible by pDST (B12). However, this mutation is flagged as a mutation with a minimum of confidence and was also called with low frequency of 6%.

### Mixed infections

The tNGS assay also generates data on mixed infections (e.g. with two *M. tuberculosis* complex strains), heteroresistance, spoligotype, and phylogenetic lineage classification. Within cohort one, four participants were found to have mixed infections (one by species identification and three by lineage-specific SNP analysis) with the minority population detected by the species identification match at 8% of the total (Additional file 1: Table S4). Multiple lineages were detected in one patient with multidrug-resistant tuberculosis. SNP-based lineage prediction was possible for 28 samples, with two identified as belonging to lineage 1, three to lineage 2, ten to lineage 4.3 and 13 which could not be further classified except as not H37Rv (Additional file 1: Table S4).

Within cohort two, two of 13 participants were found to have mixed infections (one by blast and one by lineage-specific SNP analysis) with the minority population detected by blast at 6% of the total (Additional file 1: Table S5). Overall, SNP-based lineage prediction was possible for 13 samples, with one identified as belonging to lineage 1, four to lineage 2, one to lineage 3, one with markers for lineage 1, 7 (*M. tuberculosis*), 5, 6 (*M. africanum*), animal lineages or *M. canettii* and six which could not be further classified except as being other than H37Rv (Additional file 1: Table S5).

## Discussion

In these observational cohorts of outpatients diagnosed with confirmed and probable tuberculosis in Eswatini and Germany, we demonstrate for the first time that comprehensive mDST from stool samples is possible by combining a specific DNA extraction method with targeted genome sequencing [13]. The performance and accuracy of tNGS for molecular resistance prediction from stool samples was confirmed in our validation cohort pointing towards stool as a diagnostic opportunity to complete rapid DST through tNGS when analysis in sputum fails or when sputum is not available. The data obtained also indicated that a simple pre-screening procedure based on qPCR standardized quantitative levels of *M. tuberculosis* complex DNA can be used to select samples with the highest chance of successful tNGS. This evidence highlights the potential to expand the role of stool as a specimen for the diagnosis of tuberculosis by allowing for rapid comprehensive mDST of first-line and second-line anti-tuberculosis drugs.

Stool is now recommended as a tuberculosis diagnostic specimen by the World Health Organization for use with the Xpert assay, but resistance testing with this assay is limited to rifampicin and misses relevant mutations such as I491F in *rpoB* [28]. Following a novel stool DNA extraction method [13], the tNGS assay provided sequence-based drug resistance information on 57% (41/72) of specimens positive by stool qPCR including both cohorts investigated in this study. The rate of tNGS *M. tuberculosis* complex resistance detection from stool DNA increased to 77% (34/44) when the testing was limited to specimens with a qPCR concentration of > 1 fg/μl. Similar to reductions in performance of line probe assays [29], WGS [30], and tNGS [21] with smear-negative respiratory samples, we found a reduction in *M. tuberculosis* complex resistance detection by tNGS in specimens with a qPCR DNA concentration of < 1 fg/μl. This provides a potential threshold for triaging stool samples on which tNGS can reliably be performed; thereby, reducing costs associated with unsuccessful runs.

The DNA isolation described in this study was performed in a tuberculosis research laboratory in Eswatini, proving that it can be implemented in other high-burden settings. Likewise, the Deeplex® Myc-TB assay streamlines sequencing requirements and has now been implemented in national drug resistance surveys in sub-Saharan Africa [21, 31], suggesting that this approach may also be suitable for high-burden settings. Indeed, implementation of tNGS through the SeqMDRTB_NET network project (https://ghpp.de/de/projekte/seqmdrtb-net/) in multiple Sub-Saharan countries, including Eswatini, is currently underway.

One important characteristic of the Deeplex® Myc-TB assay is the ability to interrogate 18 genomic regions involved in resistance development to 13 anti-tuberculosis drugs in clinical *M. tuberculosis* complex strains. This is crucial in areas that are affected by the epidemic spread of drug-resistant strains with resistance mutations not detected by other conventional mDST assays such as Xpert [27, 32]. For example, in Eswatini more than 50% of the multidrug-resistant *M. tuberculosis* complex strains carry the I491F mutation in *rpoB*, which is not detected by the Xpert and line probe assays endorsed by the WHO [27, 33]. As a consequence, strains with this mutation, which confers clinical resistance to rifampicin, are typically inaccurately tested sensitive by Xpert, line probe, and liquid pDST [34–37]. This leads to delayed detection of patients affected by multidrug-resistant tuberculosis, non-effective treatment, and ongoing transmission of the I491F *rpoB* outbreak strains [27, 32]. This effect is evidenced by the increase of the I491F *rpoB* outbreak strains in Eswatini, from 30% in 2008/2009 to 60% in the recent drug resistance survey [38]. Of equal concern is the fact that more than 50% of the I491F *rpoB* MDR *M. tuberculosis* complex outbreak strains also have a Rv0678 M146T mutation, which confers bedaquiline and clofazimine resistance [27]. TNGS performed directly on sputum and now on stool samples can overcome this diagnostic challenge.

The capacity to perform targeted sequencing on *M. tuberculosis* complex DNA isolated from stool also has important implications for evaluating the impact of mixed infections on patient outcomes. In this study, 12% (6/51) of patients from both cohorts with *M. tuberculosis* complex detected had evidence of mixed infections which are unlikely to be detected by liquid culture media after the growth of the predominant strain. Further studies are needed to determine to what extent tuberculosis patients are affected by mixed infection, potential differences in the detection of mixed infections in stool and sputum samples, and the impact of mixed infection on diagnostics and treatment outcomes.

Although the data presented in our study represent an important new area of research for tuberculosis diagnostics and drug susceptibility testing, our study also has limitations. As this nested study capitalized upon an existing biorepository, there may be selection bias in the samples analyzed. The sample size was modest and limited to two distinct clinical populations. Further, we could not determine whether discordance between mDST and pDST results was present due to strain differences in sputum and stool or inherent differences in molecular vs. phenotypic methods; the debate between whether the molecular or phenotypic susceptibility result should be considered the reference standard for some tuberculosis medications such as rifampicin or bedaquiline is ongoing. As *M. tuberculosis* culture performs poorly on stool, a comparison of mDST and pDST results on stool was not indicated. Finally, while these findings underline the potential impact of tNGS on stool samples as an additional diagnostic procedure, additional studies with a direct comparison of tNGS from stool and sputum will be needed to more accurately establish the target population, perhaps high-risk populations such as PLHIV and children, most likely to benefit from stool testing clinically.

## Conclusions

In conclusion, these findings represent an advance for tuberculosis diagnostics by demonstrating proof of principle that stool is a diagnostic specimen that can support rapid comprehensive mDST to inform clinicians on the choice of drugs for an individualized treatment regimen, a critically important advancement for patients with multidrug-resistant tuberculosis. The approach described in our work has the potential to increase access to comprehensive mDST for patients unable to provide sputum samples or who have greater concentrations of *M. tuberculosis* complex detected by stool PCR than in sputum specimens. In light of the rapid rollout of new treatment regimens for patients with multidrug-resistant tuberculosis, expanding access to targeted sequencing technology in high-burden settings must be a priority in the fight to end tuberculosis.

## Supplementary Information

**Additional file 1: Table S1.** Logistic regression models comparing the relationship between cycle threshold value (CT value) and femtogram per microliter (fg/ul) of MTB DNA detected by qPCR with successful detection of MTB by tNGS and successful resistance detection by tNGS. **Table S2.** Logistic regression models assessing for an association between the timing of stool collection within the study window (up to 14 days from TB treatment initiation) and detection of MTB by qPCR, successful detection of MTB by tNGS, and resistance detection by tNGS. **Table S3.** Comparison of genotypic DST vs. phenotypic DST resistance detection. **Table S4.** Mixed infection and lineage data from cohort one. **Table S5.** Mixed infection and lineage data from cohort two.

## Data Availability

The sequencing data analyzed for this study have been deposited in European Nucleotide Archive (ENA) database (Accession number: PRJEB47403, https://www.ebi.ac.uk/ena/browser/view/PRJEB47403?show=reads [25].
