# Correction: Rapid molecular diagnostics of tuberculosis resistance by targeted stool sequencing

**DOI:** 10.1186/s13073-022-01107-w

**Published:** 2022-09-20

**Authors:** Doctor B. Sibandze, Alexander Kay, Viola Dreyer, Welile Sikhondze, Qiniso Dlamini, Andrew DiNardo, Godwin Mtetwa, Bhekumusa Lukhele, Debrah Vambe, Christoph Lange, Muyalo Glenn Dlamini, Tara Ness, Rojelio Mejia, Barbara Kalsdorf, Jan Heyckendorf, Martin Kuhns, Florian P. Maurer, Sindisiwe Dlamini, Gugu Maphalala, Stefan Niemann, Anna Mandalakas

**Affiliations:** 1 National Tuberculosis Reference Laboratory, Eswatini National Health Services Laboratory, Ministry of Health, Mbabane, Eswatini; 2 Baylor College of Medicine Children’s Foundation-Eswatini, Mbabane, Eswatini; 3 Global Tuberculosis Program, Baylor College of Medicine, Houston, TX USA; 4 Department of Global Health, Amsterdam UMC, University of Amsterdam, Amsterdam, The Netherlands; 5 Molecular and Experimental Mycobacteriology, Research Center Borstel, Borstel, Germany; 6 German Center for Infection Research (DZIF), Partner Site Hamburg-Lübeck-Borstel-Riems, Borstel, Germany; 7 Eswatini National Tuberculosis Program, Ministry of Health, Mbabane, Eswatini; 8 Clinical Infectious Diseases, Research Center Borstel, Borstel, Germany; 9 Respiratory Medicine & International Health, University of Lübeck, Lübeck, Germany; 10 The National School of Tropical Medicine, Baylor College of Medicine, Houston, TX USA; 11 Cluster Precision Medicine in Inflammation, University of Kiel, Kiel, Germany; 12 Department of Internal Medicine I, University Medical Center Schleswig-Holstein (UKSH), Kiel, Germany; 13 National and WHO Supranational Reference Center for Mycobacteria, Research Center Borstel, Borstel, Germany; 14 Institute of Medical Microbiology, Virology and Hygiene, University Medical Center Hamburg-Eppendorf, Hamburg, Germany

**Correction: Genome Med 14, 52 (2022)**

**https://doi.org/10.1186/s13073-022-01054-6**

The original publication [1] contained an error in affiliations (and the order of affiliations). The article has been updated with the correct affiliations.

## References

[1] Sibandze DB (2022). Rapid molecular diagnostics of tuberculosis resistance by targeted stool sequencing. Genome Med.

